# Parental Folate Deficiency Inhibits Proliferation and Increases Apoptosis of Neural Stem Cells in Rat Offspring: Aggravating Telomere Attrition as a Potential Mechanism

**DOI:** 10.3390/nu15132843

**Published:** 2023-06-22

**Authors:** Qinghan Ren, Guoquan Zhang, Cuixia Dong, Zhenshu Li, Dezheng Zhou, Li Huang, Wen Li, Guowei Huang, Jing Yan

**Affiliations:** 1 Department of Nutrition and Food Science, School of Public Health, Tianjin Medical University, Tianjin 300070, China; renqinghan0223@tmu.edu.cn (Q.R.); zgq1217@tmu.edu.cn (G.Z.); d13516108816@163.com (C.D.); lizhenshu@tmu.edu.cn (Z.L.); dezhengzhou@163.com (D.Z.); huangli123@tmu.edu.cn (L.H.); liwen828@tmu.edu.cn (W.L.); huangguowei@tmu.edu.cn (G.H.); 2 Tianjin Key Laboratory of Environment, Nutrition and Public Health, Tianjin 300070, China; 3 Department of Social Medicine and Health Administration, School of Public Health, Tianjin Medical University, Tianjin 300070, China

**Keywords:** folate, parental, proliferation, apoptosis, telomere attrition, neural stem cells

## Abstract

The effect of maternal folate status on the fetal central nervous system (CNS) is well recognized, while evidence is emerging that such an association also exists between fathers and offspring. The biological functions of telomeres and telomerase are also related to neural cell proliferation and apoptosis. The study aimed to investigate the effect of parental folate deficiency on the proliferation and apoptosis of neural stem cells (NSCs) in neonatal offspring and the role of telomeres in this effect. In this study, rats were divided into four groups: maternal folate-deficient and paternal folate-deficient diet (D-D) group; maternal folate-deficient and paternal folate-normal diet (D-N) group; maternal folate-normal and paternal folate-deficient diet (N-D) group; and the maternal folate-normal and paternal folate-normal diet (N-N) group. The offspring were sacrificed at postnatal day 0 (PND0), and NSCs were cultured from the hippocampus and striatum tissues of offspring for future assay. The results revealed that parental folate deficiency decreased folate levels, increased homocysteine (Hcy) levels of the offspring’s brain tissue, inhibited proliferation, increased apoptosis, shortened telomere length, and aggravated telomere attrition of offspring NSCs in vivo and in vitro. In vitro experiments further showed that offspring NSCs telomerase activity was inhibited due to parental folate deficiency. In conclusion, parental folate deficiency inhibited the proliferation and increased apoptosis of offspring NSCs, maternal folate deficiency had more adverse effects than paternal, and the mechanisms may involve the telomere attrition of NSCs.

## 1. Introduction

Folate, a crucial cofactor of one-carbon unit metabolism, has been proven to be tightly associated with fetal brain formation and development due to its critical functions in the proliferation and growth of nerve cells and the synthesis of neurotransmitters [1,2]. Human data showed maternal folate concentration positively associated with fetal head growth and neurobehavioral development in the offspring [3,4]. In rodents, maternal folate deficiency may induce increased homocysteine (Hcy) transfer to the fetus, inhibit progenitor cell proliferation, increase apoptosis in the fetal forebrain, and persistently impair memory ability in offspring [5,6,7]. Recently, increasing evidence suggested that paternal folate concentration may be associated with offspring neurodevelopment. Animal research stated that paternal folate deficiency impacted placental folate transportation, DNA methylation and/or mutation, and gene expression in the neonatal rat brain [8,9]. A prospective cohort study also illustrated that periconceptional paternal folate status significantly impacted embryonic growth [10]. Existing evidence suggests that maternal and paternal folate status might be related to the development of neurological, neurocognitive, and neurobehavioral in the offspring, but the degree of influence of each maternal and paternal on offspring remains unclear.

Neural stem cells (NSCs) have the potential for multiple differentiation as well as self-renewal and generate a large number of brain cells in the central nervous system (CNS) [11]. In mammals, NSCs existed primarily in the subventricular and subgranular zones, respectively, located in the brain hippocampus’s lateral ventricle and dentate gyrus [12]. The initial stage of exerting neural functions is achieved through the proliferation of NSCs [13]. Apoptosis is an evolutionarily regulated, conserved programmed cell death that plays a critical role in normal physiological processes such as brain development and tissue homeostasis in animals, but the excessive apoptosis of NSCs has a negative impact on neurodevelopment and global brain growth [14]. Thus, regulating the proliferation and apoptosis of NSCs is crucial for CNS development [15]. In the developing CNS, the proliferation and apoptosis of NSCs are primarily influenced by epigenetic or environmental exposures (i.e., malnutrition) [16]. Craciunescu CN et al. [6] proved that maternal folate deficiency inhibited proliferation and increased apoptosis of progenitor cells in the offspring’s brain, thereby negatively affecting neurodevelopment. Our previous animal study reported that maternal folic acid supplementation during pregnancy improved NSC proliferation in rat offspring [17]. Although the necessary role of maternal folate concentration CNS development is widely recognized, the extent to which paternal folate concentration affects offspring NSCs remains uncertain. Meanwhile, the potential mechanism of parental folate deficiency affecting the proliferation and apoptosis of NSCs in offspring has not yet been revealed.

Telomere is a DNA–protein complex segment at the end of the linear chromosome in eukaryotic cells that maintains the integrity of chromosomes and the cycle of cell division [18]. Telomerase is a ribonucleoprotein enzyme synthesizing telomeric DNA to counter telomere shortening [19]. The regulation of telomeres and telomerase biological functions appears to be associated with controlling cell proliferation, differentiation, and cell death [20]. A previous study showed that telomere attrition dramatically impaired in vitro proliferation of NSCs in adult mice [21]. Additionally, evidence suggested that telomeres play important roles in regulating NSC proliferation and apoptosis and the DNA damage responses of neural cells [22]. It is reported that telomerase in NSCs influences cell proliferation, neuronal differentiation, neuronal survival, and neuritogenesis [23]. Moreover, studies have demonstrated a positive association between maternal folate levels during pregnancy and the telomere length of offspring [24]. Our previous experiments had likewise shown that folic acid inhibited NSC apoptosis in a dose-dependent manner by alleviating telomeric DNA oxidative damage and telomere attrition in vitro [25]. Therefore, it is necessary to investigate further whether parental folate deficiency affects the NSC proliferation and apoptosis of offspring by aggravating telomere attrition in NSCs.

Based on the above, maternal and paternal folate deficiency degrees influence NSC proliferation and apoptosis in offspring, and the mechanism is unclear. The present study, using an animal model of cross-generational Sprague Dawley (SD) rats, hypothesized that parental folic acid deficiency inhibits the proliferation and increases apoptosis by mediating telomere attrition of rat offspring NSCs.

## 2. Materials and Methods

### 2.1. Animals and Dietary Treatment

The experimental protocols were conducted in accordance with the ethical standards of The Tianjin Medical University Animal Ethics Committee (TMUaMEC2021027). Forty-eight female SD rats (6 w, 160–190 g) and twenty-four male SD rats (6 w, 190–230 g) were obtained from Charles River Laboratories, Beijing, China. Rats were assigned into four groups: (1) both parental feeding the folate-deficient diet (D-D) group; (2) maternal feeding the folate-deficient diet and paternal feeding the folate-normal diet (D-N) group; (3) maternal feeding the folate-normal diet and paternal feeding the folate-deficient diet (N-D) group; (4) both parental feeding the folate-normal diet (N-N) group. After two months of dietary treatment, the dams were mated with male rats with a female-to-male ratio of 2:1. The prenatal weight and conception rate of maternal rats were recorded. The rats were housed under a 12 h light/dark cycle with free access to water and food at a room temperature of 24 ± 2 °C and a relative humidity of 45–60%.

Folate-deficient diet (<0.1 mg/kg, equivalent <20 ug/d for humans) and folate-normal diet (2 mg/kg, equivalent 400 ug/d for humans) were purchased from the Trophic Animal Feed High-Technology Company (Nantong, China). After delivery, the number of offspring, the number of stillbirths, and the body weight of the neonatal rats were recorded. The neonatal rats (after birth within 24 h) were sacrificed, and the brain tissue of the pups was removed. For in vivo study, the brain tissue (cerebellum was removed) was fixed with 4% paraformaldehyde (PFA) and was processed into 3 μm paraffin sections or stored at −80 °C after liquid nitrogen flash-freezing for future assay. For in vitro study, NSCs were derived from the hippocampus and striatum tissues of newborn pups, and their parameters were measured as described below.

### 2.2. Cell Culture

The brain tissue of neonatal offspring SD rats (after birth within 24 h) was rinsed with phosphate-buffered saline (PBS), and then the hippocampus and striatum tissues were isolated and mechanically cut into 1 mm^3^ sections with ophthalmic surgical scissors and transferred to serum-free medium Dulbecco’s modified Eagle’s medium (DMEM) and nutrient mixture F-12 Ham (F12) (1:1) (Corning, New York, NY, USA) with 2% B27 (Gibco, Billings, MT, USA), 20 ng/mL epidermal growth factor (EGF; PeproTech, Cranbury, NJ, USA), 20 ng/mL basic fibroblast growth factor (bFGF; PeproTech), and 2 mmol/L L-glutamine (Sigma, St. Louis, MO, USA). Single-cell suspensions were isolated with a straw blower, seeded at 1 × 10^6^/cell/mL in 25-cm^2^ T-flasks (Corning), and cultured at 37 °C in a 5% CO_2_ atmosphere for 7 days. Purified NSCs were cultured in a medium containing different concentrations of folic acid for 7 days (D-D group and D-N group, 0 μM; N-D group and N-N group, 10 μM). After 10 days of culture under different conditions, the cells were harvested to detect proliferation and apoptosis ability, as well as telomere attrition.

### 2.3. Cell Identification

NSC neurospheres, after 7 days of purification, were inoculated on slides and incubated in a proliferative medium at 37 °C for 24 h and then fixed with 4% PFA for 20 min as well as permeabilized with 0.3% Triton X-100 at room temperature (RT). After blocking with 10% goat serum for 1 h at 37 °C, the coverslips were incubated with primary mouse anti-proliferating cell nuclear antigen (PCNA) antibody (1:100, Abcam, Cambridge, UK) and primary rabbit anti-sex determining region Y box 2 (SOX2) antibody (1:100, Abcam) overnight at 4 °C. Next, the samples were incubated with secondary antibodies (fluorescein isothiocyanate [FITC]-conjugated anti-mouse antibody, 1:100; tetramethyl rhodamine isothiocyanate [TRITC]-conjugated anti-rabbit antibody, 1:100, Sparkjade, Dongying, China) for 1 h at RT and stained with 4′,6-diamidino-2-phenylindole (DAPI) contained Vectashield (Sparkjade).

NSC differentiation was also identified similarly. NSC neurospheres were gently mechanically dissociated and plated on circular coverslips in DMEM/F12 medium containing 5% fetal bovine serum (FBS; Gibco), 2% N_2_ (Gibco) and 100 U/mL penicillin and phytomycin (Gibco) without B27, EGF, or bFGF. After 6 days of differentiation, the cell treatments were consistent with identifying the proliferation ability. The coverslips were incubated with primary mouse anti-β-III-tubulin antibody (1:100, Abcam) and primary rabbit anti-glial fibrillary acidic protein (GFAP) antibody (1:100, Bioss, Beijing, China) overnight at 4 °C. Additionally, the samples were incubated with a secondary FITC-conjugated goat anti-mouse antibody (1:100, Sparkjade) and a secondary TRITC-conjugated anti-rabbit antibody (1:100, Sparkjade) for 1 h and counterstained with DAPI (Sparkjade). Positive signals were acquired through the use of an Olympus IX81 microscope (Olympus, Tokyo, Japan).

### 2.4. Folate and Hcy Assay

The folate level in the brain tissue was measured via a competitive protein-binding assay with an automated chemiluminescence system (Siemens immune 2000 xpi, Berlin, Germany) for all types of folates with detection limits of 1–24 ng/mL. The concentration of Hcy in the brain tissue was assayed using Auto-Chemistry Analyzer (CS-T300, DIRUI, Changchun, China) with detection limits of 3–50 μmol/L. Protein quantification was assayed using bicinchoninic acid (BCA) protein quantitative Kit (Sparkjade).

### 2.5. Immunofluorescence Analysis

Brain tissue from pups was removed, and then collected and soaked in 4% PFA for 12 h, and paraffin sections were then obtained. The sections were dewaxed in xylene and incubated in an anhydrous ethanol solution. Afterward, brain sections were treated with 3% H_2_O_2_ for 10 min to block endogenous peroxidase, followed by permeabilization with 0.5% Triton X-100 for 30 min and antigen retrieval with citrate buffer at 80 °C. Next, sections were blocked with 10% goat serum for 1 h and incubated with primary mouse anti-PCNA antibody (1:300, Abcam) and primary rabbit anti-SOX2 antibody (1:300, Abcam) at 4 °C overnight. Samples were then treated with a secondary FITC-conjugated goat anti-mouse antibody (1:100, Sparkjade) and a secondary TRITC-conjugated anti-rabbit antibody (1:100, Sparkjade) for 1 h, followed by staining with DAPI. The Immunofluorescence (IF) signals were captured with an Olympus IX81 microscope and analyzed with Image J software (version 1.54, National Institutes of Mental Health, Bethesda, MD, USA).

The proliferation of NSCs in vitro was also measured via IF. Cultured NSCs were plated on coverslips and incubated in a proliferation medium containing 10 µM 10 mm 5-bromo-20-deoxyuridine (BrdU, Sigma) for 24 h, followed by fixation with 4% PFA for 30 min. After permeabilization with 0.3% Triton X-100 for 15 min and blocking with 10% goat serum for 1 h, the cells were incubated overnight at 4 °C with primary mouse anti-BrdU antibody (1:100, Sigma) and primary rabbit anti-Ki67 antibody (1:100, Abcam), respectively. After three more washes with PBS, the cells were incubated with a secondary FITC-conjugated goat anti-mouse antibody (1:100, Sparkjade) and a secondary FITC-conjugated goat anti-rabbit antibody (1:100, Sparkjade) for 1 h and then stained with DAPI. Positive cells were obtained with an Olympus IX81 microscope and analyzed with Image J software.

### 2.6. Cell Viability Assay

NSC viability was measured using the MTS (3-(4,5-dimethylthiazol-2-yl)-5-(3-carboxymethoxyphenyl)-2-(4-sulfophenyl)-2H-tetrazolium) assay with the CellTiter 96^®^ AQueous One Solution Cell Proliferation Assay (Promega Corporation, Madison, WI, USA). The cells were diluted fourfold and gently blown into the suspension and then seeded into 96-well plates at a 200 µL/well concentration. After adding 20 μL of 5 mg/mL MTS to each well, the mixture was incubated for another 3 h at 37 °C under a 5% CO_2_ atmosphere. The absorbance at 490 nm was detected using a microplate reader (ELX800uv™, Bio-Tek Instrument Inc., Winooski, VT, USA).

### 2.7. TUNEL Assay

The apoptosis of NSCs in the brain tissue was detected using TUNEL (TdT-mediated dUTP nick-end labeling) staining for the DNA fragmentation of NSCs with the TUNEL fluorescence kit (G2350, Promega). After dewaxing, hydration, immersion in 4% PFA for 15 min, and permeabilization with 20 μg/mL proteinase K solution for 10 min, 50 μL TUNEL solution was added to each slide and placed at 37 °C for 1 h. The reaction was terminated with 2× SSC (sodium citrate salt) for 15 min, and the sections were stained with DAPI. Finally, the green fluorescence (fluorescein-12-dUTP) and apoptotic cells’ blue background (DAPI) were examined under an Olympus IX81 microscope and analyzed using Image J software.

In the in vitro experiments, NSCs derived from the hippocampus and striatum tissues of the rat offspring and inoculated on coverslips after 7 days of intervention were fixed with 4% PFA for 20 min, permeabilized with 0.3% Triton X-100 for 20 min and incubated with TUNEL reaction mixture at 37 °C for 1 h. The reaction was terminated with 2× SSC for 15 min, and the cells were stained with DAPI. TUNEL-positive NSCs were identified using an Olympus IX81 microscope and analyzed with Image J software.

### 2.8. Cell Telomere Attrition Assay

The telomere length of NSCs in the hippocampus and cerebral cortex of offspring brain tissue can be measured via Q-Fluorescent In Situ Hybridization (Q-FISH) using microscopes to determine telomere fluorescence intensity after hybridization with a fluorescent peptide nucleic acid (PNA) (CCCTAACCCTAACCCTAA; F1002; PNA Bio) telomeric repeat (CCCTAA3) probe. First, paraffin sections of the brain were degreased with xylene and hydrated with gradient alcohol. After immersion in citrate buffer in a steamer until cooled at RT, the sections were permeabilized in 0.5% Triton X-100 for 30 min and then immersed in gradient ethanol at concentrations ranging from 60% to 100% for 10 min each time. The sections were treated with Cy3-labeled telomeric PNA probe hybridization solution, dried, denatured at 85 °C for 5 min, and hybridized overnight at RT for 12 h in the dark. Next, the slides were washed twice with Washing Solution I (formamide, PBS, and blocking solution) and three times with Washing Solution II (1M Tris-HCl, 3M NaCl, Tween-20, and distilled water) and then blocked with 10% golf serum and stained with primary rabbit anti-SOX2 antibody (1:300, Abcam) overnight at 4 °C. The secondary antibody was goat anti-rabbit FITC antibody (1:100, SparkJade), and finally, the samples were counterstained with DAPI. Images were taken with an Olympus IX81 microscope using a 100× objective. Telomere signal intensity was determined as PNA signal normalized with nuclear DAPI and captured by the ImageJ plugin Telometer (v2.1.4, Johns Hopkins University, Baltimore, MD, USA).

Telomere length and oxidative damage of telomeric DNA were detected via Immunofuorescence-Fluorescent In Situ Hybridization (IF-FISH) in NSCs in vitro. NSCs were previously treated as used for IF. After fixation with 4% PFA for 25 min and exposure with 0.3% Triton X-100 for 15 min, slides were exposed to Cy3-labeled telomere PNA probe hybridization solution, denatured at 85 °C for 3 min and then left in the dark at RT for 12 h for hybridization, followed by washing with a solution containing 70% formamide and 1 M Tris-HCl and a solution containing Tween-20 in Tris-buffered saline. NSCs were blocked with 10% goat serum for 1 h and then incubated with anti-phospho-histone H2AX (γ-H2AX) (Ser139) (1:500, Millipore, Burlington, MA, USA) for 12 h at 4 °C and with secondary FITC goat anti-mouse antibody (1:100. SparkJade) for 1 h. Images of cells were captured by an Olympus IX81 microscope. C-rich telomere probe-FITC (TelC-FITC) and DNA damage response protein γ-H2AX were considered telomere dysfunction-induced foci (TIF)-positive cells if there were more than two IF foci [26]. Telomere length and oxidative telomeric DNA damage were also analyzed using the Image J plugin Telometer as well.

### 2.9. Telomerase Activity Assay

Telomerase is a ribonucleic acid–protein complex composed of a single long non-coding RNA, called telomerase RNA, and associated proteins. NSCs telomerase activity was detected by Telomerase Activity Quantification qPCR Assay Kit (ScienCell, Carlsbad, CA, USA) that was amplified via quantitative real-time PCR (qPCR). Briefly, the cultured cells were lysed with lysis buffer supplemented with PMSF and β-mercaptoethanol. Then, the homogenized samples prepared were left at 4 °C for 30 min and were spun at 12,000–16,000 g for 20 min. Telomerase reactions (Cell lysate sample; 5× Telomerase reaction buffer; Nuclease-free H_2_O) were incubated at 37 °C for 3 h and terminated by heating at 85 °C for 10 min. The qPCR reactions (Post telomerase reaction sample; Primer stock solution; qPCR master mix; Nuclease-free H_2_O) were performed under the following conditions: 95 °C for 10 min, 95 °C for 20 s, 52 °C for 20 s, and amplified for 36 PCR cycles (72 °C for 45 s) with the LightCycler 480 II instrument (Roche Applied Science, Basel, Switzerland).

### 2.10. Statistical Analyses

All data were presented as mean ± SD or percentage. The chi-squared test was adopted to compare the rate distribution between groups. ANOVA performed comparisons among different groups of the variables for factorial design, and the Student–Newman–Keuls (SNK) test was used for multiple comparisons to determine significant differences among the experimental groups. The statistical analyses were performed using SPSS 24.0 (IBM Corp., Armonk, NY, USA) and *p* < 0.05 was considered statistically significant to evaluate differences between groups.

## 3. Results

### 3.1. Characteristics of Parental Rats and Offspring Rats

All maternal and paternal rats had no detectable morbidity and survived until delivery. There was a statistically significant difference in body weight of neonatal offspring between the four groups (*p* < 0.05), and no significant differences in prenatal weight, conception rate of maternal rats, and stillbirth rate of offspring between the four groups (*p* > 0.05) (Table 1).

### 3.2. Parental Folate Deficiency Decreased Offspring Brain Tissue Folate Levels and Increased Hcy Levels

**Figure 1:**
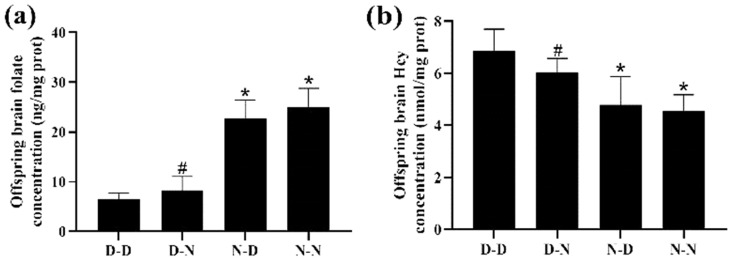
The level of folate and homocysteine (Hcy) of neonatal offspring. Rats were divided into four groups: maternal folate-deficient and paternal folate-deficient diet (D-D) group; maternal folate-deficient and paternal folate-normal diet (D-N) group; maternal folate-normal and paternal folate-deficient diet (N-D) group; maternal folate-normal and paternal folate-normal diet (N-N) group. Offspring rats were sacrificed at postnatal day 0 (PND0), and brain tissue was isolated. (**a**) The level of folate in brain tissue of PND0 offspring. (**b**) The level of Hcy in brain tissue of PND0 offspring. (*n* = 6 rats/group). *: *p* < 0.05 compared with D-D group. ^#^: *p* < 0.05 compared with N-N group.

Among the four groups, the D-D group had the lowest folate levels and the highest Hcy levels in the brain tissue of offspring rats. The N-N group had higher folate levels and lower Hcy concentration in the brain tissue of offspring than the D-N group (*p* < 0.05; Figure 1a,b). The results indicated that folate levels in the brain tissue of the offspring neonatal rats were reduced, whereas Hcy concentrations were increased by the deficiency of parental folate, and the combined effects of folate deficiency in both parents, played a more adverse part, followed by maternal folate deficiency and paternal folate deficiency.

### 3.3. Parental Folate Deficiency Inhibited Proliferation of NSCs in Hippocampus and Cerebral Cortex of Neonatal Offspring

**Figure 2:**
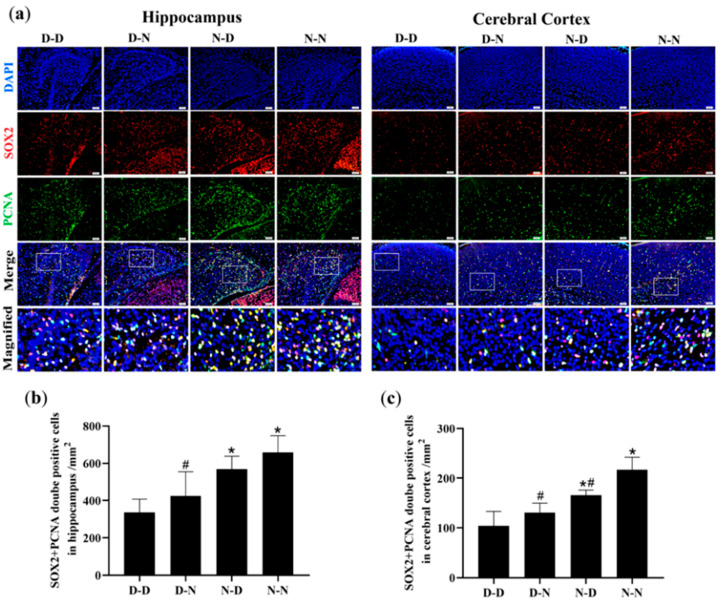
Neural stem cell (NSCs) proliferation in the hippocampus and cerebral cortex of neonatal offspring. Offspring rats were grouped, as described in Figure 1. (**a**) Representative micrographs of immunofluorescence in the hippocampus and cerebral cortex, in which proliferative NSCs are stained with proliferating cell nuclear antigen (PCNA) (green), sex-determining region Y box 2 (SOX2) (red) and 4′,6-diamidino-2-phenylindole (DAPI) (blue). Scale bar = 50 μm. Each below merge column depicts a magnified image of the rectangular region of its corresponding image in the upper merge column. (**b**) Quantitative analysis of PCNA/SOX2-double-positive cells in the hippocampus. (**c**) Quantitative analysis of PCNA/SOX2-double-positive cells in the cerebral cortex. Data are expressed as mean ± SD (*n* = 5 for each group). *: *p* < 0.05 compared with D-D group. ^#^: *p* < 0.05 compared with N-N group.

The numbers of proliferating NSCs were quantified to determine if parental folate deficiency could inhibit the proliferation of NSCs in offspring neonatal rats. Immunofluorescence analysis of the hippocampus and cerebral cortex of offspring showed that the D-D group had the fewest PCNA/SOX2-double-positive cells among the four groups, and the D-N group had fewer PCNA/SOX2-double-positive cells than the N-N group did (*p* < 0.05; Figure 2a–c). These results suggested that parental folate deficiency inhibited NSCs proliferation in the hippocampus and cerebral cortex of postnatal day 0 (PND0) offspring, with maternal folate deficiency producing more adverse impacts.

The study also investigated the regional differences in NSCs proliferation, as shown in Figure 2. The numbers of proliferative NSCs in the hippocampus were more significant than in the cerebral cortex of the offspring.

### 3.4. Parental Folate Deficiency Inhibited Proliferative Capacity and Cell Viability in NSCs of Neonatal Offspring In Vitro

To ensure whether parental folate deficiency could inhibit NSCs proliferation in vitro, NSCs derived from PND0 offspring hippocampus and striatum tissues were assessed via the use of IF with two markers, BrdU and Ki67, respectively, both of which were used to detect the proliferative capacity of the cells, while MTS was used to detect cell viability. NSC neurospheres were identified with the capacity for self-renewal as well as for neuronal and astrocytic differentiation (Figure S1).

**Figure 3:**
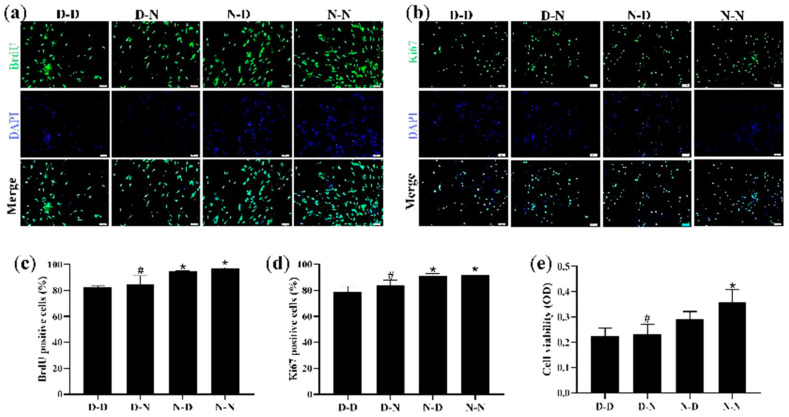
Parental folate deficiency inhibited cell viability and proliferative capacity in NSCs in vitro. Offspring were grouped as described in Figure 1, and hippocampus and striatum tissues were dissected from PND0 offspring and used to create NSCs cultures. NSC neurospheres, after 7 days of purification, were cultured in a medium containing different concentrations of folic acid (D-D group and D-N group, 0 μM; N-D group and N-N group, 10 μM) for 7 days. (**a**) Representative micrographs of the 10 mm 5-bromo-20-deoxyuridine (BrdU) incorporation assay, in which proliferative cells are stained with BrdU (green) and DAPI (blue). Scale bar = 50 μm. (**b**) Representative micrographs of the Ki67 incorporation assay, in which proliferative cells are stained with Ki67 (green) and DAPI (blue). Scale bar = 50 μm. (**c**) Quantitative analysis of BrdU-positive cells. (**d**) Quantitative analysis of Ki67-positive cells. (**e**) Cell viability detected by MTS assay. Data are expressed as mean ± SD (*n* = 5 for each group). *: *p* < 0.05 compared with D-D group. ^#^: *p* < 0.05 compared with N-N group.

The results showed that the D-D group had the fewest viable cells, fewest BrdU-positive NSCs, and fewest Ki67-positive NSCs, suggesting that parental folate deficiency caused a significant decrease in both markers (*p* < 0.05; Figure 3a–d). Meanwhile, the N-N group had higher cell viability, more BrdU-positive NSCs, and more Ki67-positive NSCs than the D-N group (*p* < 0.05; Figure 3e). These results indicated that the proliferative capacity and cell viability of NSCs in the offspring neonatal rats were reduced by parental folate deficiency, with the maternal performing a more detrimental function.

### 3.5. Parental Folate Deficiency Increased Apoptosis of Neural Cells in Hippocampus and Cerebral Cortex of Neonatal Offspring

**Figure 4:**
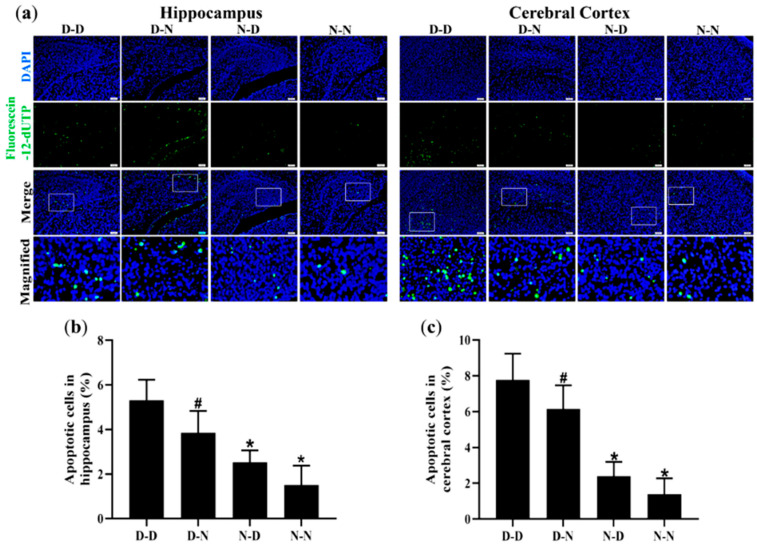
Parental folate deficiency increased the apoptosis rate of neural cells in the hippocampus and cerebral cortex of neonatal offspring. Offspring rats were grouped as described in Figure 1. (**a**) Representative micrographs of TdT-mediated dUTP nick-end labeling (TUNEL) in the hippocampus and cerebral cortex, in which apoptotic cells are stained with Fluorescein-12-dUTP (green) and DAPI (blue). Scale bar = 50 μm. Each below merge column depicts a magnified image of the rectangular region of its corresponding image in the upper merge column. (**b**) The apoptosis rate of neural cells in the hippocampus. (**c**) The apoptosis rate of neural cells in the cerebral cortex. Data are expressed as mean ± SD (*n* = 5 for each group). *: *p* < 0.05 compared with D-D group. ^#^: *p* < 0.05 compared with N-N group.

To investigate whether parental folate deficiency could increase the apoptosis of neural cells in offspring, the TUNEL assay was used to assess the apoptosis rate of neural cells in the hippocampus and cerebral cortex of offspring rats. The numbers of apoptotic cells of the D-D group were the most among the four groups, and the N-N group had fewer apoptotic cells than the D-N group did both in the hippocampus and cerebral cortex (*p* < 0.05, Figure 4a–c). Additionally, there were more apoptotic neural cells in the cerebral cortex of the brain in the rat offspring than in the hippocampus (Figure 4a,b). These results suggested that parental folate deficiency increased the apoptosis of neural cells both in the hippocampus and cerebral cortex of neonatal offspring, and the effects of maternal folate deficiency were more visible than paternal.

### 3.6. Parental Folate Deficiency Increased Apoptosis of NSCs of Neonatal Offspring In Vitro

**Figure 5:**
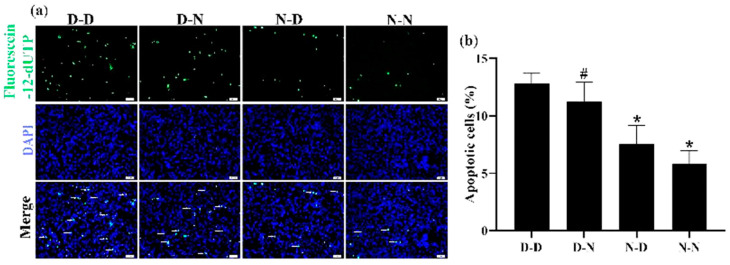
Parental folate deficiency increased the apoptosis rate of NSCs in vitro. Offspring were grouped as described in Figure 1, and NSCs were cultured as described in Figure 3. (**a**) Representative micrographs of the TUNEL assay, in which apoptotic NSCs are stained with Fluorescein-12-dUTP (green) and DAPI (blue). Scale bar = 50 μm. (**b**) The apoptosis rate of NSCs in vitro. Data are expressed as mean ± SD (*n* = 5 for each group). *: *p* < 0.05 compared with D-D group. ^#^: *p* < 0.05 compared with N-N group.

The apoptosis of NSCs derived from the hippocampus and striatum tissues of neonatal offspring was measured using the TUNEL assay to investigate further whether parental folate deficiency would increase the apoptosis of NSCs in vitro. Among the four groups, the number of apoptotic NSCs in the D-D group was the most, and the N-N group had fewer apoptotic cells than the D-N group (*p* < 0.05; Figure 5a,b). The results showed that the NSC apoptosis in vitro increased in response to parental feeding with folate-deficient diets, and maternal played a more negative role on the offspring.

### 3.7. Parental Folate Deficiency Shortened Telomere Length of NSCs in Hippocampus and Cerebral Cortex of Neonatal Offspring

**Figure 6:**
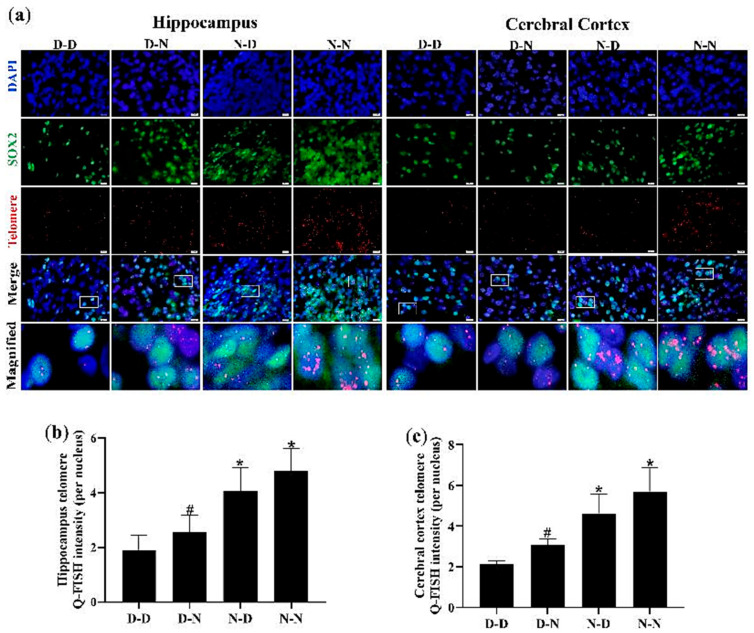
Parental folate deficiency shortened the telomere length of NSCs in the hippocampus and cerebral cortex of neonatal offspring. Offspring rats were grouped as described in Figure 1. (**a**) Representative micrographs of immunofluorescence in the hippocampus and cerebral cortex, in which NSCs were stained with SOX2 (green), telomere (red) and DAPI (blue). Scale bar = 10 μm. Each below merge column depicts a magnified image of the rectangular region of its corresponding image in the upper merge column. (**b**) Telomere length quantified using Q-Fluorescent In Situ Hybridization (Q-FISH) in the NSCs of the hippocampus. (**c**) Telomere length quantified using Q-FISH in the NSCs of the cerebral cortex. Data are expressed as mean ± SD (*n* = 5 for each group). *: *p* < 0.05 compared with D-D group. ^#^: *p* < 0.05 compared with N-N group.

To verify whether the inhibition of proliferation and the increase in apoptosis due to parental folate deficiency on offspring NSCs was associated with telomere shortening of cells, the telomere length of NSCs (SOX2-positive cells) in the hippocampus and cerebral cortex was measured using Q-FISH. As shown in Figure 6, NSC telomere length in the hippocampus and cerebral cortex was the shortest in the D-D group among the four groups. The increased telomere length of NSCs in the hippocampus and cerebral cortex of neonatal offspring was observed in the N-N group compared with the D-N group (*p* < 0.05; Figure 6a–c). These results revealed that the telomere length of NSCs in the hippocampus and cerebral cortex of neonatal offspring was shortened by parental folate deficiency. Additionally, the combined effects of folate deficiency in both parents played a more adverse part, followed by maternal folate deficiency and paternal folate deficiency. Meanwhile, these results also indicated that telomere shortening might be a potential mechanism by which parental folate deficiency inhibited cell proliferation and increased apoptosis in the NSCs of offspring.

### 3.8. Parental Folate Deficiency Aggravated Telomere Attrition and Inhibited Telomerase Activity of NSCs of Neonatal Offspring In Vitro

**Figure 7:**
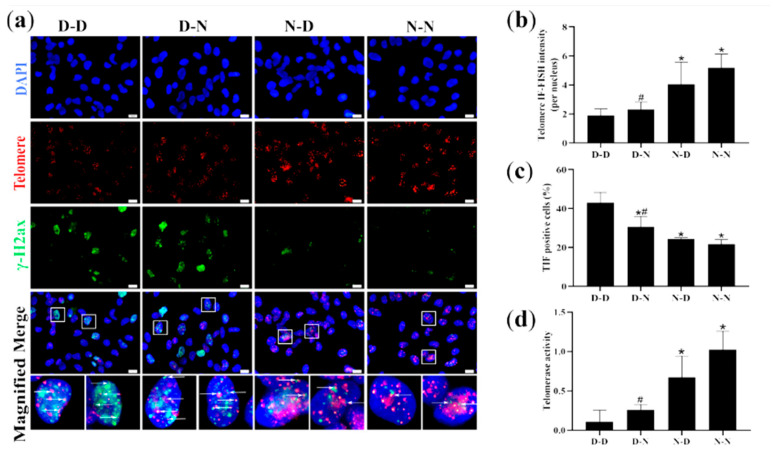
Telomere length, telomeric DNA damage, and telomerase activity of NSCs in vitro. Offspring were grouped as described in Figure 1, and NSCs were cultured as described in Figure 3. (**a**) Representative micrographs of Immunofuorescence-Fluorescent In Situ Hybridization (IF-FISH) of NSCs in vitro, in which NSCs are stained with anti-phospho-histone H2AX (γ-H2AX) (green), telomere (red) and DAPI (blue). Scale bar = 10 μm. Each below merge column depicts a magnified image of the rectangular region of its corresponding image in the upper merge column Scale bar = 10 μm. (**b**) Telomere length quantified by IF-FISH in NSCs in vitro. (**c**) Percentages of telomere dysfunction-induced foci (TIF) cells (containing both C-rich telomere probe-FITC (TelC-FITC) and γ-H2AX) of NSCs in vitro. (**d**) Telomerase activity of NSCs in vitro. Data are expressed as mean ± SD (*n* = 5 for each group). *: *p* < 0.05 compared with D-D group. ^#^: *p* < 0.05 compared with N-N group.

To ensure whether parental folate deficiency could affect telomere attrition and telomerase activity of offspring NSCs in vitro, telomere attrition and telomerase activity of NSCs derived from hippocampal and striatum tissues of offspring neonatal rats were further examined using IF-FISH and qPCR, respectively. The findings showed that the D-D group had the shortest telomere length, the most TIF-positive cells, and the lowest telomerase activity in NSCs derived from the hippocampus and striatum tissues of PND0 offspring. The N-N group had longer telomere length, fewer TIF-positive cells, and higher telomerase activity than the D-N group (*p* < 0.05; Figure 7a,d). In general, the findings demonstrated that parental folate deficiency aggravated telomere attrition and inhibited the telomerase activity of NSCs of neonatal offspring in vitro, which was more affected by maternal folate deficiency than paternal. Additionally, these results in vitro reaffirmed that parental folate deficiency might impact the proliferation and apoptosis of neonatal NSCs in the offspring by aggravating telomere attrition.

## 4. Discussion

The present results of the in vivo and in vitro experiments showed that parental folate deficiency inhibited the proliferative capacity, increased apoptosis, and shortened the telomere length of the NSCs of neonatal offspring rats. This study further provided in vitro data indicating that telomerase activity was inhibited and telomeric DNA damage was increased in NSCs derived from hippocampus and striatum tissues of neonatal offspring rats by parental low folate diets. Based on the above, the effect of parental folate status on the proliferation and apoptosis of NSCs may be mediated by telomerase activity and telomere integrity. Folic-acid-deficient diets during pregnancy could lead to negative reproductive outcomes and brain development [27]. The process of the proliferation and apoptosis of NSCs is critical for developing the brain and for plasticity and repair in the CNS [13]. Our previous study found that maternal folate deficiency inhibited the proliferation of NSCs and increased the apoptosis of neural cells in the fetal brain [17,25]. R.S. Seelan et al. [28] have also illustrated that gestational folate deficiency increased apoptosis and decreased proliferation noted in cranial neural crest cells of mice lacking folr1 (the gene encoding folate receptor 1). Although there is no direct evidence from previous studies of the relationship between paternal folate deficiency and offspring NSCs, experiments in mice have shown that low paternal folate status is associated with increased birth defects in the next generation, and paternal dietary factors influence cognitive and neural functions in the offspring mice [29]. The results of this study provided new evidence that proliferation was inhibited and that apoptosis increased in relation NSCs in offspring in response to parental folate deficiency. The effects on proliferation and apoptosis NSCs in offspring were inhibited in intensity, followed by folate deficiency of both parents, only mother and only father.

Telomeres are repetitive DNA sequences at the ends of chromosomes that prevent the loss of genomic DNA and are affected by folate [30]. An Italian population-based study reported a positive association between plasma folate concentration and telomere length [31]. Additionally, research with a sleep deprivation mouse model concluded that folic acid supplementation could effectively counteract sleep deprivation-induced telomere dysfunction and the associated aging phenotype [32]. Our previous animal studies showed that folic acid supplementation decreased uracil misincorporation in telomere, alleviated telomere length shortening, decreased neurocyte apoptosis rates, and alleviated cognitive performance in SAMP8 mice [33]. The study illustrated that parental folate deficiency shortened telomere length of NSCs in the hippocampus and cortex in neonatal offspring as well as increased telomere attrition and decreased telomerase activity in NSCs derived from the hippocampus and striatum tissues of newborn rats, providing evidence that parental folate concentration plays a vital role in telomere length and function of the offspring.

Telomeres also appear to be closely associated with cell maturation, differentiation, proliferation, and apoptosis, which is crucial for the functioning of stem cells [34]. A mouse study found that telomere shortening in NSCs disrupted neuronal differentiation and neurite formation [35]. Thanseem. I et al. [36] indicated that telomere shortening inhibited adult neurogenesis and impaired the maintenance of postmitotic neurons in the hippocampus. Apart from the maintenance of telomere length, telomerase modulates several steps in a neuron’s life cycle [34]. Telomerase was found to stimulate the proliferation of neuronal stem cells in vitro and in vivo [37], with positive effects on preventing apoptosis, promoting neurogenesis, and improving lifespan in a mouse model of neurodegenerative disease [38]. Likewise, in this study, parental folate deficiency inhibited proliferation and increased apoptosis in NSCs, and this may be mediated by shortening telomere length and reducing telomerase activity in offspring NSCs.

Folate is an essential nutrient for human health and development, and folate deficiency during pregnancy produces adverse pregnancy outcomes [39]. Adequate folate intake is important for pregnant women, who require 5–10 times more folic acid than before pregnancy to support optimal growth and development of maternal and fetal tissues [40]. Human epidemiological and animal research showed that maternal folate concentration during preconception and conception was related to the development of CNS and neurobehavior in offspring [41,42]. A prospective population-based cohort in Rotterdam, including 2095 children, showed that low maternal folate concentration during pregnancy is associated with altered offspring brain development in childhood [43]. Besides, a recent systematic review provided evidence of associations between paternal folate status and offspring health [44]; however, the effects of paternal folate levels on progeny neurodevelopment are uncertain. Animal research showed that paternal folate deficiency at mating could influence fetal brain DNA methylation and insulin-like growth factor-2 (ILGF-2) expression [45]. A meta-analysis showed that offspring rats of paternal with a folate-deficient diet are more susceptible to developing anxious and depressive traits [46] but not linked to the offspring’s neurodevelopment. Shared maternal and paternal folic acid dietary influences might potentially combine for a greater impact on later generations’ neural development. Up to now, the majority of research models have been uniparental designs, and the difference between the combined effects of folate deficiency in both parents and unilateral folate deficiency is uncharted. This experiment-induced parental folate deficiency has been found to result in a decrease in proliferation and an increase in apoptosis of NSCs in the hippocampus, cerebral cortex, and in vitro cultures of neonatal offspring, and the intensity of effect was followed by folate deficiency in both parents, only maternal folate deficiency and only paternal folate deficiency. However, the differences in proliferation, apoptosis, telomere length, and telomerase activity were not significant between the parental folate deficiency and the only maternal folate deficiency group, although a similar tendency was observed. There was a significant difference in TIF-positive cells between parental folate deficiency and only the maternal folate deficiency group.

In conclusion, the present study showed that parental folate deficiency inhibited the proliferation and increased apoptosis of NSCs in neonatal offspring rats, whereas the combined effects of folate deficiency in both parents played a more adverse part, followed by only maternal folate deficiency and only paternal folate deficiency, in which aggravated telomere attrition could serve as one of the potential mechanisms in the process. This highlights the importance of maternal and paternal folic acid supplementation during the periconceptional period.

## 5. Conclusions

The study indicated that parental folate deficiency inhibited proliferation and increased apoptosis of NSCs in neonatal offspring in vitro and in vivo, maternal folate deficiency had more adverse effects than paternal, and telomere attrition may be a mediator for this association.

## Figures and Tables

**Table 1:** Characteristics of parental rats and offspring rats.

Variables	D-D	D-N	N-D	N-N	*p*
Prenatal weight (mean ± SD)	418.69 ± 54.79	398.77 ± 42.58	429.81 ± 16.72	411.23 ± 42.42	0.755
Conception (*n*, %)	9 (75)	6 (50)	6 (50)	7 (58.3)	0.561
Offspring (*n*)	118	128	111	114	
Stillbirth (*n*, %)	4 (3.4)	10 (7.8)	6 (5.4)	11 (9.65)	0.305
Body weight of neonatal offspring (mean ± SD)	6.46 ± 0.73	6.29 ± 0.89	6.72 ± 0.75	6.43 ± 0.78	0.005

## Data Availability

The data presented in this study are available upon request from the corresponding author.

## Supplementary Materials

The following supporting information can be downloaded at: https://www.mdpi.com/article/10.3390/nu15132843/s1, Figure S1: Identification of NSCs in cell culture.
