# Supplementary material for: Parental Folate Deficiency Inhibits Proliferation and Increases Apoptosis of Neural Stem Cells in Rat Offspring: Aggravating Telomere Attrition as a Potential Mechanism
Source: Nutrients. 2023 Jun 22;15(13):2843. doi: 10.3390/nu15132843 (PMC10343379; doi:10.3390/nu15132843)
Supplement: Supplementary file 1 [file nutrients-15-02843-s001.zip › nutrients-2458226-supplementary.pdf]

Identification of NSC in Cell Culture

Hippocampus and striatum tissues were dissected from PND0 offspring and used as donors to create cell cultures in serum-free DMEM/F12 medium with B27, bFGF, and EGF. NSCs proliferated and aggregated into neurospheres during the 7-day purification (Figure S1a). Figure S1b demonstrated the cell neurospheres of the four groups on the 4th day of the intervention. As revealed by the results of the PCNA incorporation assay, almost all cells in the neurosphere were SOX2 positive and had the potential to proliferate (Figure S1c). Then the neurospheres were mechanically dissociated and the resulting cells were cultured in DMEM/F12 medium supplemented with 5% FBS and 2% N_2, but without B27, EGF, or bFGF. After 6-day culture in this differentiation medium, most of the NSCs had differentiated into neurons or astrocytes, which were identified as β-III-tubulin-positive and GFAP-positive cells, respectively (Figure S1.d). In total, these results suggest that the cultured neurospheres were comprised of NSCs with the capacity for self-renewal as well as for neuronal and astrocytic differentiation.

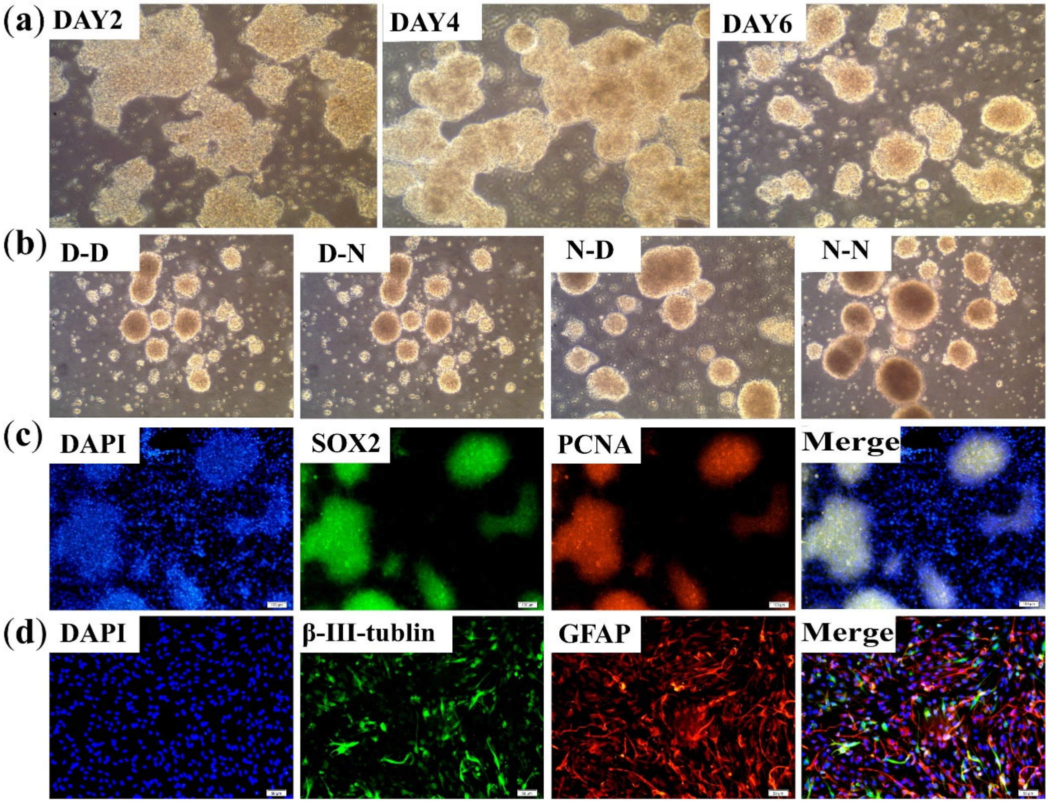

Figure S1: Identification of NSCs in cell culture. Offspring were grouped as described in **Figure 1**, and NSCs were cultured as described in **Figure 3**. (**a**) Bright field images of NSC neurospheres (100×) at various time points. (**b**) Bright field images of NSC neurospheres (100×) after seven days of intervention at various groups. (**c**) Proliferative NSC neurospheres were stained with SOX2 (green), PCNA (red), and DAPI (blue). Scale bar = 100 μm. (**d**) NSC neurospheres were dissociated and cultured in the differentiative medium, and cell nuclei were stained with DAPI (blue), neurons and astrocytes were stained with β-III-tubulin (green) and GFAP (red), respectively. Scale bar = 50 μm.
